# Ectopic pregnancy in China during 2011–2020: a single-centre retrospective study of 9499 cases

**DOI:** 10.1186/s12884-022-05269-8

**Published:** 2022-12-10

**Authors:** Haihua Xu, Guan Lin, Lifang Xue, Weifang Wu, Jinlian Ding, Chaobin Liu

**Affiliations:** 1 The Department of Obstetrics, Fujian Provincial Maternity and Children’s Hospital, Affiliated Hospital of Fujian Medical University, No. 18 Daoshan Road, 350001 Fuzhou, China; 2 The Department of Gynaecology, International Science & Technology Cooperation Base for Environmental Factors on Early Development, Fujian Provincial Maternity and Children’s Hospital, Affiliated Hospital of Fujian Medical University, No. 18 Daoshan Road, 350001 Fuzhou, China

**Keywords:** Ectopic pregnancy, Fertility policy change, Caesarean scar pregnancy, Advanced-age pregnant women

## Abstract

**Background:**

Previous studies have shown that the incidence of ectopic pregnancy (EP) is increasing in China. It is unclear, however, whether the incidence of EP has changed after the implementation of the universal two-child policy in the context of China’s aging population and declining fertility rate.

**Methods:**

Data concerning EP from January 2011 to December 2020 were collected from the hospital’s electronic medical records, which included the annual number of delivery, caesarean section rate, ectopic pregnancies, treatment of tubal pregnancy, and average costs and length of hospitalization. Trends of the EP incidence were analysed and annual percentage change (APC) was calculated using connected point regression analyses.

**Results:**

A total of 9499 cases of EP were collected, among which caesarean scar pregnancy (CSP) accounts for the second highest (6.73%). The EP per 100 deliveries revealed a downward trend, from 7.60% in 2011 to 4.28% in 2020 with an APC of -1.87 (*P* < 0.05). The maternal age was increased, especially after the implementation of the universal two-child policy. The constituent ratio for the advanced maternal age (≥ 35) and the caesarean section rate, but not the CSP, were also increased. Laparoscopic salpingectomy was the main surgical method, whereas the adoption of laparotomy and laparoscopic salpingostomy was decreasing year by year.

**Conclusions:**

Although no obvious effect of the two-child policy on EP has been observed under the conditions of this study, the change in EP especially in advanced-age women after the policy implementation needs further evaluation. A decreased caesarean section rate, in primipara is beneficial to reducing the CSP.

## Background

Ectopic pregnancy (EP) is a relatively common disorder affecting approximately 1% of all pregnancies [1], and women with the first EP are at an increased risk of adverse birth outcomes in subsequent intrauterine pregnancies [2]. In the United Kingdom, the mortality rate of EP is approximately 3.6 per 10,000 cases, while in developing countries this number can be doubled [3]. A study in the United States showed that EP increased from 11.0 to 1000 live births in 2006 to 13.7 in 2013 [4]. China is a developing country with an incidence of EP estimated at 2.5% in 2004 [5]. The number of EP cases admitted to Fudan University Obstetrics and Gynaecology Hospital, a large tertiary hospital in Shanghai, China, increased from 698 in 2003 to 1860 in 2013 [6]. After the implementation of the one-child policy in China in 1979, the total fertility rate (defined as the average number of children born per woman) was reduced from 2.9 to around 1.6 [7]. This gave rise to a delay in childbearing, as reflected by the increasing average age of puerpera from 28.1 years old in 2003 to 29.4 in 2013 in Fudan University Obstetrics and Gynaecology Hospital [6], and an increase in caesarean section rate [8].

To increase the fertility rate, China adjusted its family planning policy from the original one-child policy to a universal two-child policy in October 2015. It is not known whether such a policy change would affect the incidence of EP. The purpose of the present study is to understand the number, type, and treatment of EP in recent ten years, especially after the two-child policy implementation, especially in the context of China’s aging population and declining fertility rate.

## Methods

### Data acquisition

Data for this study came from the electronic database of Fujian Maternal and Child Health Hospital, a provincial tertiary hospital in Fujian, China, with roughly 1,293,500 outpatients, 68,800 discharges, and 21,700 deliveries in 2019. The following data from January 1, 2011, to December 31, 2020, were collected: the annual number of delivery, caesarean section rate and EP, treatment of tubal pregnancy, and average costs and length of stay in the hospital.

### Statistical analyses

Categorical variables are expressed as frequencies and percentages. Data for deliveries and EP were analysed by stratifying the age. The Connected Point Regression Program Software (Version 4.9.0.0, National Cancer Institute, USA) was used to analyse the trends. A log-linear regression model was used to evaluate the trends for annual percentage changes in the incidence of EP per 100 deliveries, the proportion of delivery and EP over 35 years old, the caesarean section rate, and caesarean scar pregnancy (CSP) per 100 deliveries. An increasing or decreasing trend was described as a positive or negative annual percentage change (APC), respectively. A two-tailed test was used, and the significance level was set at *P* < 0.05.

## Results

1. In the study period, there was a total of 9499 EP without death, of which there were 8696 cases with tubal pregnancy (accounting for 91.55% of EP), 639 with CSP (6.73%), 59 with ovarian pregnancy (0.62%), 38 with cervical pregnancy (0.40%) and 66 with other types of EP (0.69%, including abdominal pregnancy, heterotopic pregnancy, and intramural pregnancy), as shown in Fig. 1. The annual number of delivery, EP, caesarean section rate, treatment of tubal pregnancy, and average costs and length of stay in the hospital are shown in Table 1.

**Table 1:** The annual number of deliveries, caesarean section rates and ectopic pregnancies, treatment of tubal pregnancy, and averaged costs and length of stay in the hospital

Year	2011	2012	2013	2014	2015	2016	2017	2018	2019	2020
EP stratified by age, years, n(%)										
15–19	7(0.72%)	10(1.01%)	11(1.08%)	21(1.93%)	11(1.14%)	7(0.76%)	14(1.63%)	12(1.32%)	13(1.33%)	1(0.12%)
20–24	137(14.15%)	121(12.22%)	133(13.01%)	155(14.25%)	114(11.81%)	114(12.40%)	110(12.82%)	106(11.65%)	90(9.24%)	82(10.19%)
25–29	298(30.79%)	291(29.39%)	311(30.43%)	350(32.17%)	352(36.48%)	307(33.41%)	303(35.31%)	300(32.97%)	348(35.73%)	226(28.07%)
30–34	276(28.51%)	313(31.62%)	312(30.53%)	315(28.95%)	275(28.50%)	296(32.21%)	243(28.32%)	272(29.89%)	309(31.72%)	288(35.78%)
35–39	201(20.76%)	180(18.18%)	201(19.67%)	205(18.84%)	165(17.10%)	148(16.10%)	150(17.48%)	161(17.69%)	161(16.53%)	156(19.38%)
≥ 40	49(5.06%)	75(7.58%)	54(5.28%)	42(3.86%)	48(4.97%)	47(5.11%)	38(4.43%)	59(6.48%)	53(5.44%)	52(6.46%)
Number of EP	968	990	1022	1088	965	919	858	910	974	805
Number of CSP	30	65	73	69	80	77	43	63	73	66
Delivery stratified by age, years, n(%)										
15–19	94(0.74%)	75(0.51%)	81(0.60%)	60(0.40%)	108(0.71%)	71(0.44%)	37(0.22%)	63(0.35%)	71(0.33%)	67(0.36%)
20–24	1893(14.86%)	1819(12.29%)	1651(12.14%)	1383(9.30%)	2020(13.31%)	1627(10.01%)	1293(7.69%)	1392(7.82%)	1724(7.96%)	1366(7.27%)
25–29	5915(46.44%)	6844(46.26%)	5927(43.58%)	6564(44.15%)	7013(46.19%)	7176(44.13%)	6827(40.61%)	7503(42.14%)	9092(41.99%)	7300(38.85%)
30–34	3503(27.50%)	4367(29.52%)	4203(30.90%)	4850(32.62%)	4380(28.85%)	5186(31.89%)	5530(32.90%)	5802(32.59%)	7521(34.73%)	7143(38.01%)
35–39	1108(8.70%)	1394(9.42%)	1437(10.57%)	1620(10.90%)	1442(9.50%)	1890(11.62%)	2621(15.59%)	2465(13.85%)	2722(12.57%)	2482(13.21%)
≥ 40	223(1.75%)	296(2.00%)	302(2.22%)	389(2.62%)	219(1.44%)	311(1.91%)	503(2.99%)	578(3.25%)	523(2.42%)	433(2.30%)
Number of deliveries	12,736	14,795	13,601	14,866	15,182	16,261	16,811	17,803	21,653	18,791
Caesarean section rate	41.24%	40.78%	38.88%	34.80%	34.19%	34.27%	36.57%	36.76%	35.91%	38.94%
Treatment of tubal pregnancy										
A Hospitalization days	8.95	8.13	8.47	9.23	8.48	7.84	6.68	6.82	5.89	5.85
A Averaged costs($)	NA	NA	NA	1536.95	1827.64	1817.43	1702.47	1699.07	1647.02	1543.50
B Hospitalization days	7.57	7.37	7.25	7.99	7.85	7.14	7.68	7.27	6.22	6.94
B Averaged costs($)	NA	NA	NA	1434.67	1583.53	1553.09	1638.02	1661.36	1552.63	1572.74
C Hospitalization days	9.60	9.27	8.36	8.78	16.00	10.00	8.25	11.50	10.40	0.00
C Averaged costs($)	NA	NA	NA	1559.65	2334.25	2053.93	1631.23	1572.75	1720.83	0.00
D Hospitalization days	15.29	13.94	12.46	13.64	12.89	10.70	11.98	10.39	10.64	9.87
D Averaged costs($)	NA	NA	NA	500.80	555.48	539.35	630.99	600.54	594.50	540.48

A: laparoscopic salpingectomy, B: laparotomy, C: laparoscopic salpingostomy, D: methotrexate and expectant treatment. NA: unavailable data for the treatment costs prior to 2013

*EP* Ectopic pregnancy, *CSP* Caesarean scar pregnancy

**Fig. 1:**
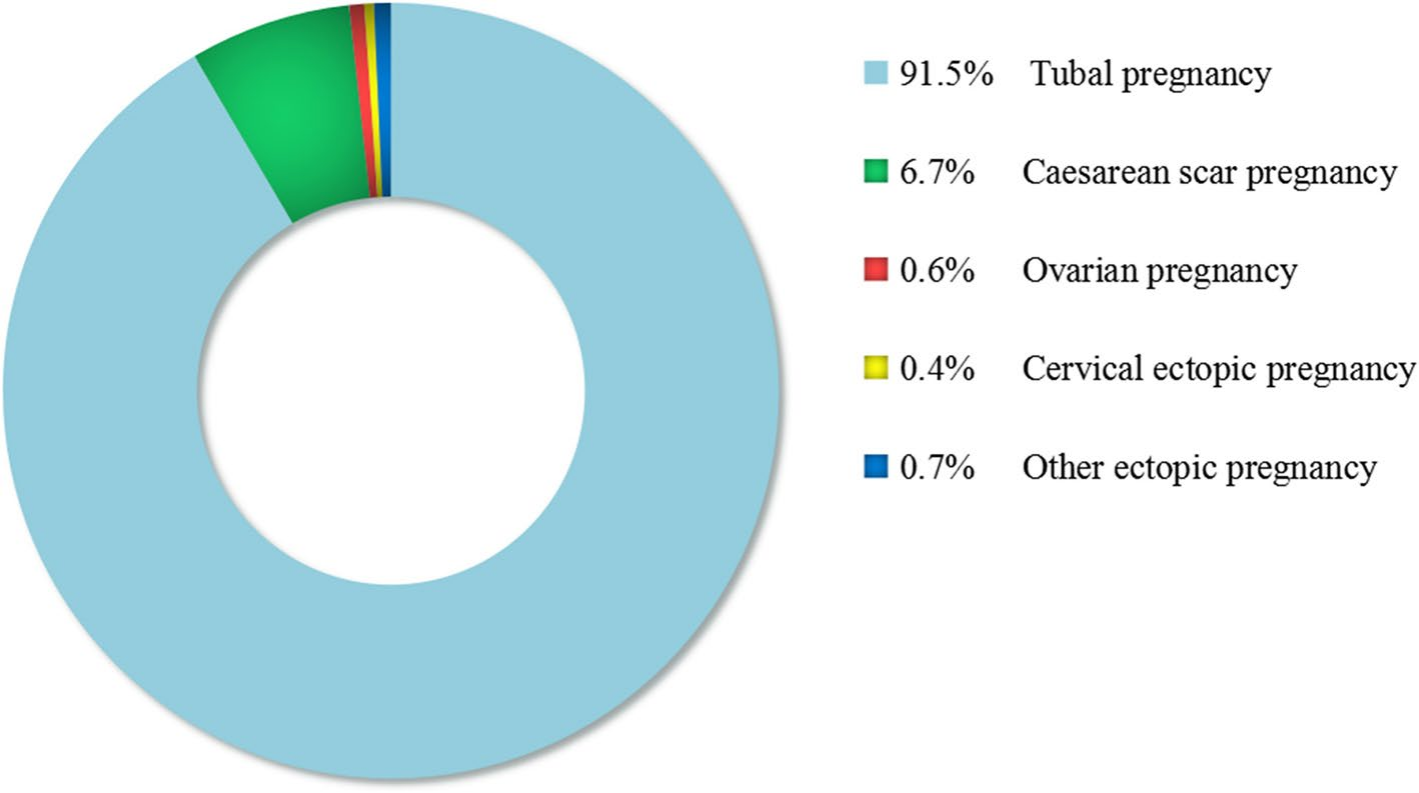
Subtypes and proportions for ectopic pregnancy
2. The annual number of EP showed a downward trend from 968 in 2011 to 805 in 2020, and EP per 100 deliveries also showed a downward trend from 7.60% in 2011 to 4.28% in 2020 with an APC of -1.87 (*P* < 0.05). Trends for the proportion of delivery and EP in the women over 35 years old, and the caesarean section rate, except CSP per 100 deliveries, were significantly different when compared between the two time periods approximately before and posterior to the two-child policy implementation, as seen in Table 2.

**Table 2:** Join point trends in the proportions of delivery and ectopic pregnancy over 35 years old, caesarean section rate and caesarean scar pregnancy per 100 deliveries

Period	Proportion of delivery in mothers over 35 years old	Proportion of EP in women over 35 years old	Caesarean section rate	CSP per 100 deliveries
APC(Trend 1)	6.94*(2011–2018)	-4.13*(2011–2016)	-5.22*(2011–2015)	45.42(2011–2013)
APC(Trend 2)	5.12(2018–2020)	4.04(2016–2020)	2.36(2015–2020)	-7.95(2013–2020)

An increasing or decreasing trend was described as a positive or negative APC, respectively. *: Statistical significance at *P* < 0.05.

**Fig. 2:**
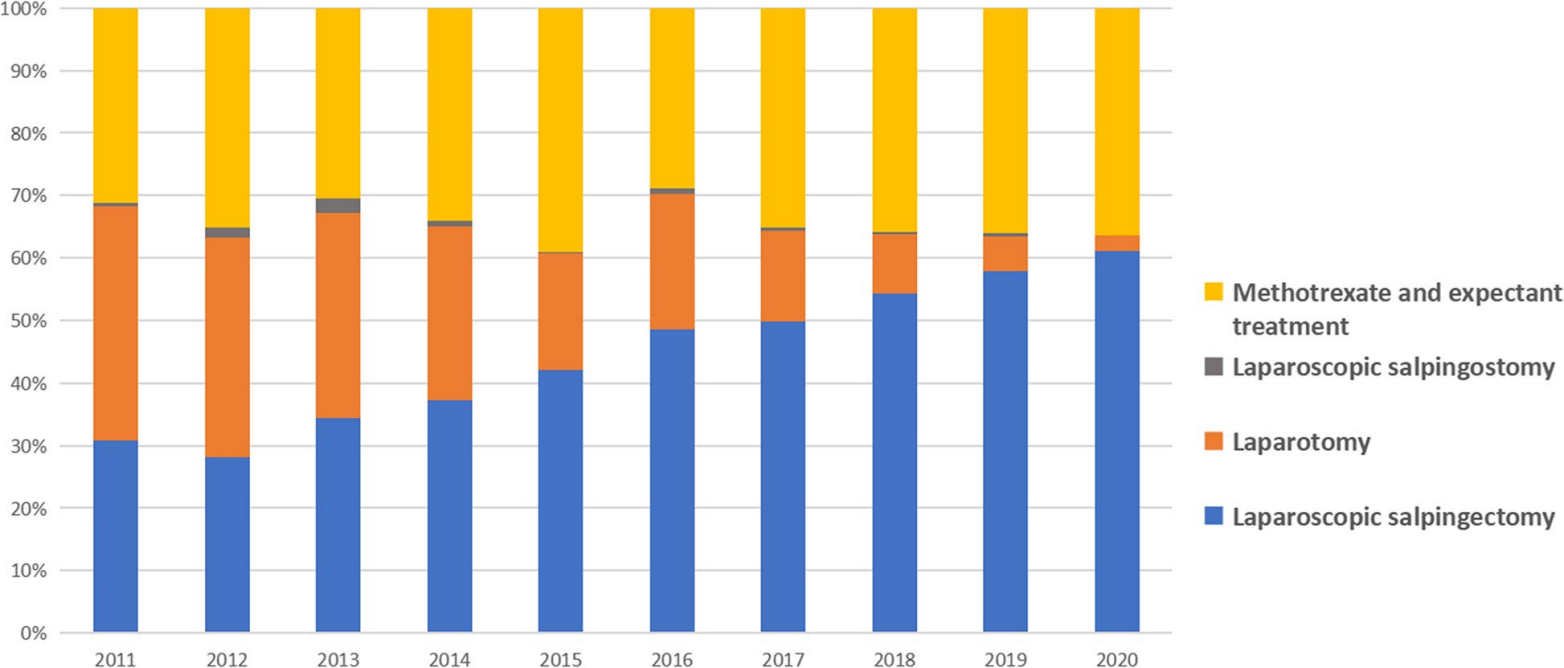
Ten-year changes in the proportion of tubal treatment methods

*APC *Annual percent change, *EP* Ectopic pregnancy, *CSP* Caesarean scar pregnancy
3. During 2011–2020, 66% of the women with tubal pregnancies were treated surgically, and 34% non-surgically. Laparoscopic salpingectomy was the main surgical approach, whereas laparotomy and laparoscopic salpingostomy were decreasingly used year by year. The proportion of the four treatment methods for tubal pregnancy, i.e., methotrexate and expectant treatment, laparoscopic salpingectomy, laparotomy, and laparoscopic salpingostomy, is shown in Fig. 2.

4. Curettage was performed in 90% of CSP under ultrasound guidance within 24–48 h of uterine artery embolism. Evaluation for the risk of major bleeding from the uterine artery was performed before curettage. Laparoscopic or transabdominal local lesion resection and uterine repair were conducted in the remaining 10% of CSP.

## Discussion

In the present study, the overall EP incidence showed a downward trend in China during 2011–2020, possibly resulting from the popularization of contraception and the successful control of sexually transmitted diseases [9]. Contraceptive measures can reduce EP [10]. From 1980 to 2010, the use of contraceptives in China, including intrauterine devices, oral contraceptives, and condoms, remained at the highest level in the world, e.g., a prevalence rate of as high as 89.20% in 2010 [11], although the contraceptive rate among women aged 15–49 declined from 86.6% in 2014 to 80.6% in 2017 after the implementation of two-child policy [9]. Sexually transmitted diseases, which have been effectively controlled in China, can increase EP [12]. Although syphilis and gonorrhea showed an increasing trend from 2014 to 2019 globally, the incidence rates of syphilis and gonorrhea were 38.37 and 8.45 per 100,000 person-years in China in 2019 [13, 14], which are far lower than the global incidences of 178.48 and 1124.39 per 100,000 person-years, respectively, in 2019 [15]. Incidence of human immunodeficiency virus infection and acquired immune deficiency syndrome has also declined in China over the past two decades, with a prevalence rate of human immunodeficiency virus infection at 39 per 100,000 person-years compared to 476 per 100,000 person-years globally in 2019 [16].

The maternal age of women with EP or delivery was mainly distributed at 25–29 and 30–34 as seen in Table 1. However, the number of women with EP or delivery aged 25–29 declined while those aged 30–34 rose after 2016, resulting in the number of EP in women aged 30–34 slightly exceeding that of EP in women aged 25–29 years by 2020. The progressive increase in the proportion of EP and births in women aged 30–34 years may be related to late marriage and changes in family planning policy. Since the implementation of the two-child policy, the proportion of advanced-age mothers for childbearing has increased significantly [17], as indicated by the increase from 8.52% in 2013 to 15.82% in 2017 in Zhejiang Province, China [18]. In the present study, we found that the proportion of advanced maternal age increased from 2011 to 2018, whereas the proportion of EP among those ≥ 35 years old showed a decreasing tendency from 2011 to 2016, which was subsequently reversed to an increasing tendency from 2016 to 2020 after the release of the two-child policy in 2015, despite the absence of a statistical significance for the latter.

After the release of the two-child policy in 2015, the proportion of EP among those ≥ 35 years old was reversed from a downward trend ( 2011–2016 APC − 4.13*) to an upward trend (2016–2020 APC 4.04), which suggests that this policy change may have an impact on EP. It is reported that the two-child policy implementation may be correlated with the decreased contraceptive rate [9], increased gonorrhea and syphilis [13, 14], and increased advanced maternal age, all of which are the possible causes for the increase of advanced EP [6, 17, 19]. Given that the short observation period, limited sample size, inadequate geographical representation and negligence of co-effects by the potential confounders might have compromised our results, which possibly makes the upward trend not statistically significant, the impact of the two-child policy on EP needs further investigation.

A single-centre study in France reported that tubal pregnancy accounted for 95.5%, ovarian pregnancy for 3.2%, intra-abdominal pregnancy for 1.3%, and no cervical and caesarean scar pregnancy (CSP) among the 1800 cases of EP from 1992 to 2001[20]. Our study also showed that tubal pregnancy accounted for the majority of 91.55% in our centre from 2011 to 2020. CSP, which refers to the gestational sac attached to the scar part of the previous uterine incision when the patient is pregnant again after the caesarean section. [21], is one of the potential long-term complications of caesarean section. Harb et al [22] concluded that the prevalence of CSP among pregnant women was about 1.5/10,000 pregnancies in the United Kingdom between 2013 and 2015. Maymon et al [23] reported that CSP accounted for 4.2% of patients with EP in a large tertiary hospital in Israel between 2000 and 2009. In China, Jiao et al [24] reported that CSP accounted for 1.05% of EP cases in Peking Union Medical College Hospital from January 1994 to April 2007, and the ratio for CSP versus total pregnancy was 1:1221. CSP in our hospital accounted for 6.73% of EP, with a ratio versus deliveries at 1:254. The prevalence of CSP in our study was highest among those described in the literature mentioned above, which could be attributed to the higher prevalence of caesarean section. [9], as well as the improvements in diagnostic ultrasound and heightened awareness of the condition by the clinician. In addition, the discrepancy also resulted from the comparison by using different denominators including the number of deliveries or the total number of pregnancies, which biases the results.

Our study revealed a decreasing trend for CSP from 2013 onwards, contributing somewhat to the decline in overall EP. The overall caesarean section rate also showed a decreasing trend from 2011 to 2020, consistent with the results from other studies in which a decreased caesarean section rate was found in all age groups, especially in younger women who had a greater decrease than older ones [25]. In addition, studies have found a decreased caesarean delivery rate in primigravida women [17]. Whereas the two-child policy was implemented in 2015, the increased number of high-risk pregnant women, such as women with multiple pregnancies, recurrent caesarean sections, and women beyond 35 years old, contributed to the resurgence of caesarean section. [17, 25]. This subset of high-risk individuals may be unwilling to experience an additional pregnancy. The decrease in the overall caesarean section rate, especially among young age and primiparous women, might contribute to the decreased trend for CSP.

For the management of tubal pregnancies, 66% were treated surgically and 34% non-surgically in our hospital during 2011–2020. Our data are close to those reported by Taheri [26], in which surgery accounted for 62% and non-surgical treatment for 38%.

The constituent ratio for the patients receiving medical therapy remained comparatively high and increased from 31.22% in 2011 to 36.43% in 2020. This may be partly because of the increasing awareness of pregnancy and the development of medical technology to diagnose ectopic pregnancy much earlier, providing early-stage patients more opportunities to receive medical treatment. It is also possible that some of the patients had less desire to accept the removal of their fallopian tubes because of the loosened childbearing policy and preferred more to medical therapy for later pregnancy. In addition, the financial problem might be another reason to choose non-surgical therapy for a few patients. A relatively high proportion of medical treatment versus surgery may also be due to the referral centre status.

The operation method for tubal pregnancies was principally laparoscopic salpingectomy in our hospital, and laparotomy and laparoscopic salpingostomy was significantly reduced, which is consistent with the report by an Australian team [27]. A reduction in open surgery is encouraged as the development of surgical techniques and the preference for small scars. Laparoscopic salpingostomy is acceptable to gynecologists and patients owing to the existence of a normal contralateral fallopian tube and comparable reproductive outcome compared to laparoscopic salpingectomy, although it brings a risk of bleeding and persistent or recurrent EP [28]. Our data showed a short-time but high-cost hospitalization for surgical treatment compared to non-surgical treatment. Although non-surgical treatment has a lower cost and can avoid the potential dangers of surgery, it needs to be carefully evaluated because approximately 15% of the patients with tubal pregnancy receiving methotrexate due to ectopic obstruction or rupture eventually require surgery [29].

CSP can be treated in a variety of ways, including curettage, surgical removal of the lesion (via abdomen or vagina), medical therapy (local or systemic injection of methotrexate), and expectant management. Almost all CSPs were surgically treated in our hospital, which may be attributed to a large number of the cases that suffered from relatively severe conditions in tertiary hospitals. Curettage was performed in 90% of the cases with pre-evaluation for the risk of major bleeding from the uterine artery before curettage, and laparoscopic or transabdominal local lesion resection and uterine repair were conducted in 10% of the cases. Harb et al. [22] reported that 61% (56/92) of CSP were treated surgically, 16% (15/92) medically, and 23% (21/92) by expectant management; the authors proposed substantial benefits of surgical therapy, including a high success rate, low complication rate, and short post-treatment follow-up. However, there have been no consensus and standard treatment for CSP thus far, and the choice of surgical treatment or non-surgical therapy is mainly based on the patient’s and local medical conditions, which may also require a combination of multiple treatment modalities for some patients.

There are limitations in this observational study. The study was a single-centre, retrospective cross-sectional study, and some clinical and missing data could not be assessed. The number of women enrolled and the geographical area covered in the study are limited, which could result in potential bias. In addition, we cannot establish a cause-effect relationship. Given that our hospital is the largest medical unit of Obstetrics and Gynaecology in Fujian Province, China, serving a diverse urban and rural population and diversified ethnics with a large delivery number of over 20,000 cases annually, it does not adequately represent the circumstances throughout the country. The total number of women at childbearing age should have been used as the denominator for the incidence of EP. Unfortunately, we failed to capture the pregnancy trends that changed over time in this way. Although the ratio/ratio calculation method used in this study has its limitations, live birth or deliveries is a suitable indicator for the comparison of EP when the number of cases enrolled increases with time [4, 30].

## Conclusion

The proportion of EP among those ≥ 35 years old was reversed to an upward trend after the release of the two-child policy in China in 2015, which may suggest a possible impact of the policy change on EP. It is of significance to continue the evaluation of the relations between changes in China’s fertility policy and EP in the future. A more stratified analysis of the population with EP is needed, and more detailed studies on the identification of high-risk factors for EP and reproduction after EP are required.

Declined incidence of EP and improvements in surgical modalities have made the treatment of EP overall promising. The popularization of contraception, the control of sexually transmitted diseases, age-appropriate pregnancy, and the reduction of the caesarean section rate are all conducive to reducing EP; In particular, a decreased caesarean section rate of primipara is helpful to reduce CSP.

## Data Availability

All data related to this study are available from the corresponding author upon reasonable request.

## Abbreviations

EP: ectopic pregnancy
APC: annual percentage change
CSP: caesarean scar pregnancy

## References

[1] Al Naimi A, Moore P, Brüggmann D, Krysa L, Louwen F, Bahlmann F (2021). Ectopic pregnancy: a single-center experience over ten years. Reprod Biol Endocrinol.

[2] Chouinard M, Mayrand MH, Ayoub A, Healy-Profitós J, Auger N (2019). Ectopic pregnancy and outcomes of future intrauterine pregnancy. Fertil Steril.

[3] Rouse CE, Eckert LO, Babarinsa I, Fay E, Gupta M, Harrison MS, et al. Spontaneous abortion and ectopic pregnancy: case definition & guidelines for data collection, analysis, and presentation of maternal immunization safety data. Vaccine. 2017;35(48 Pt A):6563–74. 10.1016/j.vaccine.2017.01.047. 10.1016/j.vaccine.2017.01.047 PMC5714431 29150062

[4] Mann LM, Kreisel K, Llata E, Hong J, Torrone EA (2020). Trends in ectopic pregnancy diagnoses in United States Emergency Departments, 2006–2013. Matern Child Health J.

[5] US Census Bureau. Various years. Available at http://www.census.gov/programs-surveys/international-programs.html. Accessed 22 Sept 2020.

[6] Li XL, Du DF, Chen SJ, Zheng SH, Lee AC, Chen Q (2016). Trends in ectopic pregnancy, hydatidiform mole and miscarriage in the largest obstetrics and gynaecology hospital in China from 2003 to 2013. Reprod Health.

[7] Hesketh T, Zhou X, Wang Y (2015). The end of the one-child policy: lasting implications for China. JAMA.

[8] Long Q, Kingdon C, Yang F, Renecle MD, Jahanfar S, Bohren MA (2018). Prevalence of and reasons for women’s, family members’, and health professionals’ preferences for cesarean section in China: a mixed-methods systematic review. PLoS Med.

[9] Qiao J, Wang Y, Li X, Jiang F, Zhang Y, Ma J (2021). A Lancet Commission on 70 years of women’s reproductive, maternal, newborn, child, and adolescent health in China. Lancet.

[10] Schultheis P, Montoya MN, Zhao Q, Archer J, Madden T, Peipert JF (2021). Contraception and ectopic pregnancy risk: a prospective observational analysis. Am J Obstet Gynecol.

[11] Wang C. Trends in contraceptive use and determinants of choice in China: 1980–2010. Contraception. 2012;85(6):570–79. 10.1016/j.contraception.2011.10.014. 10.1016/j.contraception.2011.10.014 22176789

[12] Coste J, Laumon B, Brémond A, Collet P, Job-Spira N (1994). Sexually transmitted diseases as major causes of ectopic pregnancy: results from a large case-control study in France. Fertil Steril.

[13] Yue X, Gong X, Li J, Zhang J. Epidemiological trends and features of syphilis in China, 2014—2019. Chinese Journal of Dermatology. 2021;54(8):668–72. 10.35541/cjd.20210098.

[14] Yue X, Gong X, Li J, Zhang J. Epidemiological trends and features of gonorrhea in China, 2015–2019. Chinese Journal of Dermatology. 2020;53(10):769–73. 10.35541/cjd.20200623.

[15] Zheng Y, Yu Q, Lin Y, Zhou Y, Lan L, Yang S (2022). Global burden and trends of sexually transmitted infections from 1990 to 2019: an observational trend study. Lancet Infect Dis.

[16] Govender RD, Hashim MJ, Khan MA, Mustafa H, Khan G (2021). Global epidemiology of HIV/AIDS: a resurgence in North America and Europe. J Epidemiol Glob Health.

[17] Li H, Xue M, Hellerstein S, Cai Y, Gao Y, Zhang Y, et al. Association of China’s universal two child policy with changes in births and birth related health factors: national, descriptive comparative study. BMJ. 2019;366:l4680. 10.1136/bmj.l4680. 10.1136/bmj.l4680 PMC6699592 31434652

[18] Zhang X, Chen L, Wang X, Wang X, Jia M, Ni S (2020). Changes in maternal age and prevalence of congenital anomalies during the enactment of China’s universal two-child policy (2013–2017) in Zhejiang Province, China: an observational study. PLoS Med.

[19] Ankum WM. Higher maternal age was associated with increased risks for fetal death and ectopic pregnancy. Evid Based Med. 2001;6(1):28. 10.1136/ebm.6.1.28.

[20] Bouyer J, Coste J, Fernandez H, Pouly JL, Job-Spira N (2002). Sites of ectopic pregnancy: a 10 year population-based study of 1800 cases. Hum Reprod.

[21] Timor-Tritsch IE, Monteagudo A, Calì G, D’Antonio F, Kaelin Agten A (2019). Cesarean scar pregnancy: diagnosis and pathogenesis. Obstet Gynecol Clin North Am.

[22] Harb HM, Knight M, Bottomley C, Overton C, Tobias A, Gallos ID (2018). Caesarean scar pregnancy in the UK: a national cohort study. BJOG.

[23] Maymon R, Svirsky R, Smorgick N, Mendlovic S, Halperin R, Gilad K (2011). Fertility performance and obstetric outcomes among women with previous cesarean scar pregnancy. J Ultrasound Med.

[24] Jiao L, Zhao J, Wan X, Liu X, Feng F, Ren T, et al. Diagnosis and treatment of cesarean scar pregnancy. Chin Med Sci J. 2008;23(1):10–5. 10.1016/S1001-9294(09)60002-X. 10.1016/s1001-9294(09)60002-x 18437903

[25] Liang J, Mu Y, Li X, Tang W, Wang Y, Liu Z (2018). Relaxation of the one child policy and trends in caesarean section rates and birth outcomes in China between 2012 and 2016: observational study of nearly seven million health facility births. BMJ.

[26] Taheri M, Bharathan R, Subramaniam A, Kelly T (2014). A United Kingdom national survey of trends in ectopic pregnancy management. J Obstet Gynaecol.

[27] Paull C, Robson SJ (2018). Hospital admission and surgical approach to tubal ectopic pregnancy in Australia 2000 to 2014: a population-based study. Aust N Z J Obstet Gynaecol.

[28] Mol F, van Mello NM, Strandell A, Strandell K, Jurkovic D, Ross J (2014). Salpingotomy versus salpingectomy in women with tubal pregnancy (ESEP study): an open-label, multicentre, randomised controlled trial. Lancet.

[29] Sowter MC, Farquhar CM, Petrie KJ, Gudex G (2001). A randomised trial comparing single dose systemic methotrexate and laparoscopic surgery for the treatment of unruptured tubal pregnancy. BJOG.

[30] Olamijulo JA, Okusanya BO, Adenekan MA, Ugwu AO, Olorunfemi G, Okojie O (2020). Ectopic pregnancy at the Lagos University Teaching Hospital, Lagos, South-Western Nigeria: temporal trends, clinical presentation and management outcomes from 2005 to 2014. Niger Postgrad Med J.

